# Human development I: Twenty Fundamental Problems of Biology, Medicine, and Neuro-Psychology Related to Biological Information

**DOI:** 10.1100/tsw.2006.153

**Published:** 2006-07-06

**Authors:** Tyge Dahl Hermansen, Søren Ventegodt, Erik Rald, Birgitte Clausen, Maj Lyck Nielsen, Joav Merrick

**Affiliations:** ^1^ Quality of Life Research Center, Teglgårdstræde 4-8, DK-1452 Copenhagen K, Denmark; ^2^ Research Clinic for Holistic Medicine, Denmark; ^3^ Nordic School of Holistic Medicine, Copenhagen, Denmark; ^4^ Scandinavian Foundation for Holistic Medicine, Sandvika, Norway; ^5^ Interuniversity College, Graz, Austria; ^6^ Vejlby Lokalcenter, Vejlby, Denmark; ^7^ National Institute of Child Health and Human Development, Israel; ^8^ Center for Disability and Human Development, Faculty of Health Sciences, Ben Gurion University, Beer-Sheva, Israel; ^9^ Office of the Medical Director, Division for Mental Retardation, Ministry of Social Affairs, Jerusalem, Israel

**Keywords:** quality of life, QOL, holistic biology, clinical holistic medicine, public health, theoretical biology, Denmark

## Abstract

In a new series of papers, we address a number of unsolved problems in biology today. First of all, the unsolved enigma concerning how the differentiation from a single zygote to an adult individual happens has been object for severe research for decades. By uncovering a new holistic biological paradigm that introduces an energetic-informational interpretation of reality as a new way to experience biology, these papers will try to solve the problems connected with the events of biological ontogenesis involving a fractal hierarchy, from a single cell to the function of the human brain. The problems discussed are interpreted within the frames of a universe of roomy fractal structures containing energetic patterns that are able to deliver biological information. We think biological organization is guided by energetic changes on the level of quantum mechanics, interacting with the intention that again guides the energetic conformation of the fractal structures to gain disorders or healthiness. Furthermore, we introduce two new concepts: “metamorphous top down” evolution and “adult human metamorphosis”. The first is a new evolutionary theory involving metamorphosis as a main concept of evolution. The last is tightly linked to the evolutionary principle and explains how human self-recovery is governed. Other subjects of special interest that we shall look deeper into are the immunological self-nonself discrimination, the structure and function of the human brain, the etiology and salutogenesis of mental and somatic diseases, and the structure of the consciousness of a human being. We shall criticize Szentagothais model for the modulated structure of the human cerebral cortex and Jernes theory of the immunological regulatory anti-idiotypic network.

## INTRODUCTION

Several well-known phenomena in the living world have not yet found an adequate explanation. What is human consciousness and how is it connected to the brain and the wholeness of the organism? How is the differentiation of the cells brought about in the developing organism during ontogenesis? Basically, how does a fertilized egg develop into an adult organism? What are the forces behind the evolution of the living world? How is biological information stored and distributed in the organism – is everything really in the DNA? How is information managed in a living organism? How does the phenomenon of self-nonself discrimination, the basis of immune system regulation, arise? How does an organism maintain and re-establish biological order and, thus, health and physical well-being during its lifetime? What are the structural relations of the living world with the physical energy of the world's particles, atoms, and molecules? And what happens when the cellular order is disturbed and suddenly reappears in spontaneous healing? We shall address these persistent questions in the next paper devoted to the unsolved topics and discuss their relevance for clinical holistic medicine.

These questions and many more have been discussed by the research team of the Quality of Life Research Center in Copenhagen over the past years, but during the last year, work has been done to put the thoughts, questions, and discussions into paper form and relate them to clinical holistic medicine. There will be 20 papers in this series that will discuss a theory for life, biological information, healing, and adult human metamorphosis for use in clinical holistic medicine.

Biology, medicine, and neural sciences have fundamental problems that are related to the fundamental problem of biological information. This problem in science can be worded simply: What is life?[1] This is how basic it is. Everything about life is mysterious: its joy, its wisdom and tacit knowledge and competence, its ability to assume form and to be creative and intelligent, and of course, its fundamental ability to be conscious. The mystery of the two sides of life, the objective material and structural side, and the subjective experiential problem might be the hardest of all the problems. This problem is often referred to as *the hard problem*[2]. Penrose, Hameroff, and others have addressed the hard problem using the concepts, formalisms, and ideas from quantum mechanics. The problem with using concepts from physics is that physics has as many fundamental problems as does biology, and many physical theories are in conflict these days, such as the theory of relativity and the theory of quantum mechanics. Niels Henrik David Bohr (1885–1962) stated that you should not make pictures of the quantum levels of energy, because quantum mechanics does not explain about reality, but only something about how we describe reality, which has made it difficult to proceed. We believe that physics should provide us with pictures and understanding of the energy of the universe, but this has not happened yet. The supersymmetrical strings (superstring theory), wormholes, etc. have provided us with some pictures and ideas of the energy of the world, but are still not usable for biology. In 1996, Holger Bech Nielsen (professor at the Niels Bohr Institute in Copenhagen), Søren Ventegodt, and the Dalai Lama had a discussion on "science and spirituality", where the Dalai Lama explained that when his friend meditated he could make knots on hard needles. Holger Beck Nielsen concluded that the whole physics had to be changed if that was true and he has often said (as member of the study group "Matter, Life and Consciousness") that he doubts if consciousness really exists because there are no laws in physics that make this kind of integrative and nonlocal phenomenon possible.

For many years, brain research has been unable to explain the most simple things like memory and perception. Actually, the analysis into feature detectors in the brain have made the problem on qualia much more obvious. Qualia is the basic quality of perception (like the color red), which the brain really cannot provide, as the neurons that make the brain use signals that are simple electrical impulses without any quality attached to them, according to contemporary physical and biochemical laws! Biology is full of fundamental problems, like the sad fact that chemistry cannot provide the spatial information to build an embryo according to the known laws of chemistry and diffusion, as we are going to demonstrate in the papers in this series.

When this is said, nobody can be surprised that complicated things like cellular order of the body, morphogenesis, evolution and phylogenesis, perception and psychosis, disease and healing are not understood.

In this introductory paper, we want to list some of the problems that we are going to address and give our solution to. Hopefully, after reading the 20 papers in this series, you will actually have an idea of biological information, consciousness, health, disease, and healing. If we succeed in our mission and make at least some physicians develop an understanding of phenomena like spontaneous healing and human adult metamorphosis, we can hope to have strengthened the general belief in the patient's inner healing powers. This understanding is important in order to mobilize in the patient the all-important fight against unhappiness, disease, and poor ability.

In these 20 papers, we would like to address some of the following fundamental issues and problems of holistic biology:

- There are many fundamental problems in biology, medicine, and neuropsychology; most of these problems are related to biological information.
- The problem of science is that the disciplines use different concepts and methods, making it very difficult to establish the needed interdisciplinary and integrated theory for matter, life, and consciousness. Without such a multidisciplinary approach, there is little hope ever to get to an understanding of complex phenomena like healing and human adult metamorphosis.
- We have a body-mind and brain-mind, each carrying its own perspective of life and reality. How can we unite these almost opposite and contradicting interpretations of the world into one wise synthesis?
- The living cell cannot be understood through the concept of self-organization because more information is needed than the molecules apparently can provide. And consciousness remains a riddle.
- Biochemistry cannot provide the spatial information needed to explain morphogenesis.
- Supracellular morphogenesis is mysterious. Nobody seems to understand the origin of biological and cellular order.
- Physical energy is deeply mysterious. How can we proceed when the physicist will not give us the explanatory tools we need as biologists?
- Quantum chemistry could be the key to cell consciousness. Can a deeply structured quantum field give cells memory, coherence, intent, consciousness, and choice?
- What is the wholeness of man? How can 10,000 billion cells say they are me?
- Evolution has bred a fly with eyes and humans with a large wonderful brain. But how did the organisms materialize these things? Is creative metamorphosis a product of the conscious wishes of plants, animals, and humans?
- How can we understand the structure of the cerebral cortex, the most complex structure in the known universe? Can we reduce it to something simple like a number of modules in the brain?
- How does the brain think? Can it think without the body? Can it think without the wholeness (including the soul)? How does it remember? How does it organize a model of reality?
- Can a fractal music theory based on holistic brain theory explain the foundation of minor major?
- We survive by degenerating our existence. Can adult human metamorphosis upgrade our purpose of life, our level of existential responsibility, and the depth of our consciousness if we are low lifes?
- Why do we get mentally ill?
- How can the etiology of mental diseases be explained by the mental and spiritual crises of metamorphosis?
- How is the immune system regulated?

- How does the immune system function? How does the immune system make self-nonself discrimination?
- Why do we get physically ill?
- What is healing and how can we get well again when we are ill? What is spontaneous healing? Is human adult metamorphosis really a possibility?

## 20 PAPERS ON HUMAN DEVELOPMENT AND HOLISTIC BIOLOGY

### I. Twenty Fundamental Problems of Biology, Medicine, and Neuropsychology Related to Biological Information

In this current paper, we have listed all the problems touched on in this series of 20 papers. Here we make an overview of the papers to make it easier to look over the knowledge accumulated in the papers. This provides the possibility to draw out all the knowledge desired by each reader, and thereby use the papers as a work of reference.

### II. We Need an Integrated Theory for Matter, Life and Consciousness to Understand Life and Healing

We have experimented with supporting the philosophical development of patients to induce spontaneous healing. Recently, we have observed a pattern of rapid spontaneous healing that seemingly can facilitate the spontaneous recovery of cancer and mental diseases like schizophrenia and depression. Our working hypothesis is that the accelerated healing is a function of the patient's brain-mind and body-mind coming closer together due to the development of what we call "deep" cosmology. In this paper, we will explain the theory behind adult human metamorphosis and spontaneous healing.

### III. Bridging Brain-Mind and Body-Mind. Introduction to "Deep" (Fractal, Poly-Ray) Cosmology

The rational/mental and the emotional/intuitive interpretation of reality are often in fundamental conflict with each other. In order to obtain a truer knowledge, we have to form a synthesis between these two interpretations of reality. Hereby the rational and the emotional interpretations are transformed into a new, deeper interpretation cleansed of the idiosyncrasies of both mind and feelings.

In this paper, we will introduce a synthetic way of thinking needed to understand life better. It introduces our holistic biological perspective, which we call an energetic-informational interpretation of reality. In the energetic-informational interpretation of reality, bodies and particles lose their status as separate entities and are replaced by patterns of energy and information that cannot be definitely separated from the wholeness they constitute. The theory of superstrings[3] empowers such thinking; here, energy and information are conceived as being small vibrating "strings" able to form all kinds of particles. The energy of the world has holistic qualities that guide the holistic structures of the living world.

### IV. The Living Cell has Information-Directed Self-Organization — and Consciousness

This paper will discuss the ontogenesis of the cell. The DNA processing to proteins will be discussed as a system that is not able to transfer the information necessary to organize the proteins in the cell, but only to form the shape of the proteins. We will describe the structure of the information-carrying field recruited by the cells that we think is responsible for building the organelles. We use the cell's superior control of its cytoskeleton as an example of how the cell is using an informational field of positional information to guide the cell movement. We will propose that ontogenesis can be explained by *information-directed self-organization* of cells and that a superior control of the cytoskeleton through positional information lies behind cell movement. We also discuss the shape of organelles and find that self-organizing proteins cannot form these. In this paper, we will try to solve the mystery of what lies behind the mechanical cause and nature of biological information.

## V. Biochemistry Cannot Explain Morphogenesis; We Need Something Else!

To understand what the cell is, it is important to understand what it is not. We do not think the cell is a kind of "controlling machine" that produces proteins with the capability of organizing the information for the biological ontogenesis into completely functional structures through genetic control. In this paper, we will argue that the simple gradient model proposed for the morphogenesis of protostome animal lines does not find their parallel with the deuterostome lines; chemical gradients do not explain the biological functions of vertebrates. We do not think the cell is only a chemical machine. The point of view today is that information to the organization of living beings, as well at cell level as superior levels, is committed to the genes[4], but we do not find the morphological organization by DNA and simple self-organization capable of explaining the biological organization through the morphogenesis. In this paper, we will argue that the chemical gradient model is not able to explain the ontogenesis. We argue that "Turing structures" are able to describe a simple biological system only and why this model is not able to explain the complexity of biological phenomena.

## VI. Supracellular Morphogenesis — the Origin of Biological and Cellular Order

All biological systems have systemic information, their source of morphological information, in their complex dynamic[5]. During the ontogenesis and morphogenesis of the body from one cell to a multicellular organism, the biological information manifests itself through positional information. The biological system is able to develop its form and maintain it throughout its life, even in spite of lesions because there exists a system of information-transmitting interactions that is constantly adjusting all the parts of the organism to each other and to the basic blueprint. This paper takes its starting point in the subjects of morphogenesis and ontogenesis of biological organisms, and is a condition for understanding biological aspects in the view of our propose for the superior structure of nature represented in paper VII[6]. The development of the complexity of biological systems will be discussed in radical terms under the concepts of the ontogenesis. In this paper, we will discuss examples such as, for instance, regeneration and induction of the body form. We will describe the morphogenesis as a complex bifurcation where the different levels are organized in a fractal manner. We think that the development of the nerve system and other complex matters can only be described by positional information. We will argue that our model is able to explain all kinds of distribution of information through morphogenesis and ontogenesis.

## VII. A Spiral Fractal Model of Fine Structure of Physical Energy Could Explain Central Aspects of Biological Information, Biological Organization, and Biological Creativity

By introducing degrees of freedom to a system, it is possible to characterize its dimensions. In an abstract sense, these dimensions (i.e., the dimensions of space) may be thought of as freedom degrees; the more dynamic an interpretation of reality we need, the more degrees of freedom are needed, and we therefore search for a model that exceeds the normally allowed 3+1 dimensions of space and time. We propose a simple breakdown of space from 3+1 to an infinite number of dimensions. This is possible if we describe space-time itself as a spiral fractal yielding a large number of curvilinear dimensions convoluted within each other, acting at many levels. Each of these dimensions have some freedom to contain their own organizations and patterns, but they also share information with adjacent dimensions; but movement is in a particular sense one-directional, as all the dimensions are ordered in a row, so a pattern must move from one dimension to an other in order, thus giving rise to time.

However, these patterns are also subjected to mutual interactions at all levels, making the structure "magical" and unpredictable. Here we represent the mathematical-physical basis of our new paradigm. Everything can be explained as manifestation of information materializing the organization of the organism and its ecosystem. The structure of the universe thus provides *information-directed self-organization* to the ontogenesis, morphogenesis, and continuous existence of the living organism, maintaining its biological order through life of both body and mind, including its consciousness, feelings, love, sex, social activities, and using talents at work.

## VIII. A Theory of "Deep" Quantum Chemistry and Cell Consciousness. A Deeply Structured Quantum Field can Give Cells Memory, Coherence, Intent, Consciousness, and Choice

Quantum chemistry governs all activity in a biological body from biophysical and biological activity to genetic control. The collected energetic processes of a body form a superorbital that, through quantum mechanical activity, governs all processes for an organism to gain fitness and survival. This energetic quantum activity, from all the electron orbitals in the wholeness of a particular body, forms the superorbital of the individual organism and is the source of the consciousness. The more complex an organism is, the higher level of united energies the body represents, and the more "intelligent" the organism has the capability of being. All fractal levels that are involved in forming an organism, e.g., the human body, involve all superorbitals gathered on this specific level. This means that all levels have their own superorbital, e.g., the superorbital of a cell, an organ, a tissue, and the body as a whole and all superorbitals from downwards levels are gathered in the superior-level superorbitals. In this way, the human superorbital responsible for sourcing the human consciousness is formed by the gathered energies of the body as a wholeness. And "deep" quantum chemistry is the source of the human consciousness and this deeply structured quantum field gives the body cells memory, coherence, intent, consciousness, and the capability of choice.

The background for such structural activity is the fractal structure that explains the ontogenesis as a function of positional information organized as energetic particles (energetic superstrings) distributed throughout each specific fractal level and elevating downwards fractal levels to superior levels gradually with the development when higher structures arises. These energetic superstrings are gathered to energetic fields representing all energies of an organism.

## IX. A Model for the Wholeness of Man, His Consciousness and the Collective Consciousness

When the brain's simplified interpretation of the world dominates in man's perceptual representation of the totality, the result is the rational/mental interpretation of reality. On the other hand, when the direct representation outside the brain dominates the perceptual representation, we have the emotional/intuitive interpretation of reality[5]. The double representation of the world is a condition for our sound ability to be a conscious part of the world. Most aspects of man's conscious functioning can seemingly be explained by *information-directed self-organization* of the mind and of the information system of the organism at large: awareness, perception, will, consciousness, intent, morality, intuition, and spirituality. Understanding that consciousness is bound to the organism and all its relations, and not only to its nervous system, opens up for a completely new perspective of the faculties of human consciousness. It also sets the function of the brain in perspective[7].

## X. Explanation of the Macro-Evolution: Top Down Evolution Materializes Consciousness. The Origin of Metamorphosis

In the evolution of life, the possibilities provided by the inner coherence of life and of the outer physical environment of the earth are utilized to a higher and higher degree; actually the world of the living beings is mostly created by the organisms themselves, working together in a highly intelligent way (the Gaia Hypothesis[8]). From the first organisms to man, the emphasis has shifted from ecological adaptation to the environment, over mutual adaptation to the intentions of other species, to man's shaping of the organic world using his will and intention. Behind the origin of life and the course of evolution is a driving, organizing force that we think is linked to the fine structure of the universal energy itself; it seems like the wholeness has an urge for manifesting all the complexity and "wonders" of the spiral fractal. This is similar to a creative God in many religions. We will discuss the macro-evolution as a function of coherence and *information-directed self organization* in the complex and dynamic global ecosystem.

In this paper, we will discuss the "metamorphous top down" evolution in the view of the new holistic fractal paradigm. The "Metamorphous top down" evolution is a new evolutionary theory, where a top down development interacts with Charles Darwin's "bottom up" evolution, and an evolutionary event that guides such shift makes the possibility of biological diversity by the creation of varieties and new species through a metamorphous development of traits that favor survival in a specific habitat or microhabitat. We will discuss this top down model of evolution as materialized consciousness by intended metamorphosis.

## XI. The Structure of the Cerebral Cortex: Are there Really Modules in the Brain?

Many theories of the function of the neocortex are grounded on a structure, where the cortex is built up by discrete modules as described on the basis of work by the neuroanatomist Janos Szentagothai (1912–1994)[9]. However, evidence does not seem to exist concerning modules in his model, as approximately 300-μ large, discrete columns making up the association-cortex's "building stones". We will discuss examples and argue that there is no significant evidence that supports his model. As arguments for the cortex-module, we present the limited information of genes, the corticocortical afferents termination, and the columns in primary sensory cortex. From this, we conclude that evidence for the existence of the postulated module or column does not exist in the mentioned material.

## XII. A Theory for the Structure and Function of the (Human) Brain

The concept of *information-directed self-organization* gives a special meaning to the function of the brain. The brain is basically connected to itself, to the outer world through the senses, to the body though the nerves, and to the wholeness through the coherence given from the fundamental structure of the world. When interpreting the world, the wholeness of the organism constructs a meaningful whole using both emotional/spiritual and mental/cognitive intelligence. This is in contrast with the common notion that the perception itself takes place in the brain. We think that consciousness is a function of the whole organism, not a phenomenon that can be explained by brain activity alone. A double, simultaneous representation of the world is required for a perception to give meaning[5]. In this paper, we will propose a new model for the function of the brain, making good sense, if we go back to the evolutionary line and study the first nervous systems[10]. We believe the organism represents the outer world in this way: directly through the wholeness of the organism, it makes sense of the world using conscious coherence; it uses this sense or feeling/intuition to organize the perception of reality in the brain by *information-directed self-organization.* What seems to be a simple interpretation of reality through the senses is actually a highly complex creation using much more information that just what come through the eyes and ears. The organism's wholeness administers the highest intentions of the organism, giving rise to the organism's fundamental interpretation of the world. At one basic level, the brain administers events in time and space, so we can share a common reality, but as soon as we dig one step deeper, we find that interpretation of the world is highly individual. Only when the physician understands the unique model of the world in each patient, the patient can be helped to improve his quality of life and health.

## XIII. Music Theory Based on Holistic Brain Theory: Minor-Major Explained

In this paper, we will show how beautiful music can be formed on the basis of the scales of major and minor. It is discussed how the minor scale can be formed by a fractal structure as a kind of downwards reflection of the major scale to form the minor scale. It is discussed how the most beautiful and the most ugly tones are formed, and how the most beautiful tones may be behind the formation of the human recognition of music. We conclude that the simplest sound is the source of the beautiful tones and the most complex is the source of the ugly tones. To get a better understanding of the analysis and give an introduction of the different concepts used in this paper, we will discuss the concepts of sound, rhythm, and tone as foundations of the music. The relationship between wavelength, frequency, the cent intervals of the sound, and the extended system of overtones and the intervals will be discussed, and we will present an expansion of our system concerning tonic dynamics and the exploration of the complex overtone-multidimensionality, all things of a main importance for formation of music.

## XIV. Degeneration and Regeneration of Existence — Metamorphosis Changes the Purpose of Life, the Level of Existential Responsibility, and the Depth of Consciousness (the Ray-Number of the Cosmology)

We believe that Pavlovian conditioning, which is basically learning by means of rewards and punishments, through childhood, and "self-conditioning", which is the child's active adaptation to a dysfunctional family, can cause both mental[5,11] and physical[5] diseases. The process of learning in the sense of gaining knowledge seems to be quite the opposite process of learning through the conditioning process, which seems to make children more primitive in behavior and less emotionally intelligent[12]. What happens in general in the pathogenic process is that the person is losing his existential coherence because he needs to repress his character and purpose of life to get love and understanding from his parents. When the purpose of life (which is also the primary talent of the person[13]) is lost, the sound and simple way to understand and live life is also sadly lost. To heal his existence, the patient needs to rehabilitate the existential coherence though his regaining of the ability to perceive the world as it is, to be able to once again establish the synthesis of both the rational/mental and the emotional/intuitive interpretation of reality. By this process, the person's degenerated intent can be revived and brought back into use in accordance with his true biological nature.

This healing process of integration between the rational/mental and the emotional/intuitive interpretation of reality takes place when the patient takes a moment to go through life's often emotionally painful events. This process systematically reorganizes the information stored in the organism, the wholeness is cleaned of negative attitudes and conceptions, and the sound and natural original intent (which we often call the purpose of life) is regenerated. This understanding of the process of existential healing seems to be in accordance with the principles of salutogenesis described by Aaron

Antonovsky (1923–1994)[14] and of our own clinical holistic medicine[15,16,17]. We will also propose an explanation of the mind-body problem ("the hard problem") and give our solution to the problem of free will vs. determinism.

## XV. The Biochemical Hypothesis for the Etiology of Mental Diseases is not Substantiated

The nature of conditionings is suppression of the natural biological unfolding and growth, resulting in degeneration of the original, constructive intention of the individual, the purpose of life[13]. Conditioned people will, whenever suppressed emotions and feelings of love are needed later in life, experience an insurmountable wall of suppression, which results in some degree of holding back or even depression. Neurosis is the outcome of the parent's conditioning of the child[11]: the degenerate intentions of the parents are built into the model of reality in the brain of the child. Schizophrenia can be seen as a result of the person objecting to living its own life, that is, massive self-destruction or self-repression. We think this is caused by conflicts between the emotional and the rational interpretation of reality within the child, resulting in self-denial and loss of motivation. Unsolved conflicts through childhood and the consequence of following the rational/mental and not the emotional/intuitive interpretation of reality is the topic of this paper, where we will discuss the etiology of mental diseases; we will also discuss the evidence from psychoneuropharmacology for the biochemical hypotheses of schizophrenia.

## XVI. General Etiology of Mental Diseases and the Role of Remetamorphosis in Spontaneous Recovery ("Monster Theory")

Mentally ill patients can enter the state of adult human metamorphosis in order to redo the juvenile metamorphosis into nonhuman forms that was necessary for their survival in childhood. At first glance, adult human metamorphosis looks like a normal psychotic crisis of a schizophrenic patient, but when studied closer, it becomes obvious that it is an unusual state of accelerated human self-healing. It is extremely important to understand that adult human metamorphosis is the inverted event of a juvenile metamorphosis, where the patient turned him/herself into some nonhuman alien form in order to survive. The transport of existence into an alienation of existence brings severe developmental problems and both physical and mental problems to the person later in life. But this transformation was emotionally necessary for survival. We have by now carefully observed several cases of spontaneous remission of a number of different mental diseases induced by holistic existential therapy: schizophrenia, borderline schizophrenia, anxiety, and bulimia. We understand the adult human metamorphosis as a confirmation of our hypothesis of etiology of mental diseases: that they are in general caused by juvenile metamorphosis. We believe that in cracking this "secret code", we have found a general way to understand and heal mental diseases, by taking the patients into human adult metamorphosis with clinical holistic medicine.

## XVII. Jerne's Anti-Idiotypic Network Theory Cannot Explain Self-Nonself Ddiscrimination

This paper will discuss the immunological network theory by Niels K. Jerne (1911–1994)[18]. He tried to explain the immune system's capability to discriminate between self and nonself at a systemic level, but he assumed the process to be mechanical. We believe it to be directed by the same level of the organism that gives emotions and consciousness. We find little experimental evidence to support Jerne's theory, but fair empirical support for our own hypothesis.

## XVIII. A Theory for Immune System Regulation (the "Self-Nonself Discrimination")

Biological systems that have the ability to distinguish self from nonself seem to be the source of immunological reactions in higher animals, but self-nonself discrimination is found in simpler organisms as well. The organism's systemic information enables its cells to "perceive" whether a structure in the organism is self and fine, or non-self and not fine. When a structure or process of an organism at any level is in deep accordance with the blueprint of the organism materialized through the *information-directed self-organization* of the cells and organs, these structures and processes are perceived as "self" and accepted. When this part of the organism and its life are in focus, the feeling is good. When a structure or process is in conflict with the blueprint of the organism, this is perceived as nonself and it is connected with negative emotions. Self-nonself discrimination works on all levels of the organism, from the structure of the organs to its consciousness, behavior, and even sexuality. The world is divided into good and bad, wanted and unwanted, on all levels from physiology to sociology and further out to the global ecological level.

The immunological, the morphological, and the mental self-nonself discrimination thus have the same systemic source (the informational system of the organism at large) and are thus not independent of each other. In this paper, we will discuss the complex dynamic of the self-nonself discrimination as the key factor in the immunology. When self-nonself discrimination breaks down as seen in autoimmune diseases, what happens is that the ability of the organism to react toward the non-self-antigens and to refrain from reacting toward self-antigens breaks down. We suggest that the cause of such a disturbance of the immunological self-nonself discrimination is not to be found at the cellular level, but at the supracellular, systemic level. In this paper, the immunological self-nonself discrimination will be discussed in the view of the holistic paradigm.

## XIX. The Etiology of Somatic Diseases and the Physical and "Energetic" Crises of Metamorphosis

Here we give our explanation of the nongenetic, nontraumatic, somatic diseases' general etiology. We hypothesize these diseases to be caused by informational disorder giving problems to the organism's cells, disturbing them so that they become unable to function normally and do what they are supposed to do in the body. For most diseases, the etiology is unknown; we find the following three causes in general: (1) genetic disorders like DNA mutations and chromosomal defects, (2) traumatic action from a physical influence that overpowers what the healthy body can resist, and (3) biological and cellular disorders caused by informational disturbance influences the cells and organ systems. We assume that all three causes are relevant in almost all diseases; this is why we suppose that most diseases that lack obvious genetic or traumatic causes can be understood as failure in the organisms *information-directed self-organization*. This disturbance is explained by resignation and repression of the person's basic intentions (the purpose of life) and a general symptom of this sad state of being is a mechanical and nonemotional interpretation of reality. The documented occurrence of the placebo effect and the general meaning of the psychical factor in almost all diseases[19,20] indicates a huge importance of the third etiological cause, a part of the emotional reality interpretation. In this view, we will discuss the etiology of the somatic diseases seen in the perspective of our model, and we will discuss the conditioned learning as resulting in forced self-nonself choices in opposition to the biological intentions of the organism. Most nongenetic, nontraumatic somatic diseases like allergic reactions and abnormal cell (i.e., cancer) growth can be explained as caused by a disturbed informational system of the organism.

## XX. A Theory for Spontaneous Healing: Adult Human Metamorphosis

In this paper, we will present a model for adult human metamorphosis, discuss its genetics, the therapeutic tools that induce it, the relation between complexity of cosmology and spontaneous metamorphosis, spontaneous healing of cancer after metamorphosis, induced recovery from schizophrenia with metamorphosis, body reshaping after metamorphosis, and shamanism and drug-induced adult human metamorphosis.

Personal development, development of talents, spontaneous healing, personal transformation and many more forms of personal growth seems to be a kind of human metamorphosis. Survival behavior from early childhood gives us these irrational perceptions and behaviors. Such patterns seem to come from "downgrade" human metamorphosis. Therefore, adult human metamorphosis seems to be the efficient medicine most patients need to heal. Hallucinogenic drugs can induce human metamorphosis, as the Native Americans have used during many centuries, and we see the use of such drugs among young people as their attempt to get support to their healing and could suggest that these drugs should be legalized for careful medical use. We recommend experimental testing if such drugs, combined with training in deep cosmology, can induce recovery from schizophrenia, other mental diseases, and cancer. We warn against giving sedative drugs to patients during the process of metamorphosis because this will disturb the process of healing. Instead, we recommend attentive and loving care as the best medicine.

## ACKNOWLEDGMENTS

These studies were supported by grants from IMK Almene Fond. Our research in quality of life has been approved by the Copenhagen Scientific Ethical Committee under number (KF)V.100.2123/91.

## BIOSKETCHES

**Tyge Dahl Hermansen**, **MSc** in biology, is a research assistant at the Quality of Life Research Center, Copenhagen. Research areas include molecular biology, physiology, and life quality; experimental experience in fruit flies, yeast, bacteria, and cnidarians; theoretical research in human life quality; technical knowledge in the areas of animal physiology, molecular biology, biotechnology, biochemistry, cell biology, and immunology. Publications appear in popular scientific as well as international journals. E-mail: TD@livskvalitet.org

**Søren Ventegodt, MD, MMedSci,** is the director of the Nordic School of Holistic Health and Quality of Life Research Center in Copenhagen, Denmark. He is also responsible for a Clinical Research Clinic for Holistic Medicine in Copenhagen and is a popular speaker throughout Scandinavia. He has published numerous scientific or popular articles and a number of books on holistic medicine, quality of life, and quality of working life. His most important scientific contributions are the comprehensive SEQOL questionnaire, the very short QOL5 questionnaire, the integrated QOL theory, the holistic process theory, the life mission theory, and the ongoing Danish Quality of Life Research Survey, 1991–94 in cooperation with the University Hospital of Copenhagen and the late professor of pediatrics, Bengt Zachau-Christiansen, MD, PhD. E-mail: ventegodt@livskvalitet.org. Website: www.livskvalitet.org

**Erik Rald, MSc** in biology, is a research assistant at the Quality of Life Research Center, Copenhagen. His research areas and publication topics include systematics of acalypterate Diptera; history and epistemology of ecology, evolution, and taxonomy; nature preservation in nature parks; systematics, ecology, and chemistry of agaric Fungi; conservation of endangered species; assessment of nature quality in grasslands, woodlands, and urban areas. E-mail: erik_rald@scientist.com Website: http://www.dknatur.dk

**Birgitte Clausen, RN,** leader of a secluded ward for people with dementia. Dementia coordinator and consultant in Aarhus, Denmark with more than 10 years of experience in adult psychiatry and dementia. During the last 4 years, she has worked together with Søren Ventegodt on the “QOL as medicine project” and on developing existential holistic group therapy. Through her articles and lectures, she has disseminated “The New Culture of Dementia” in both Denmark and Japan. She has participated in the production of several videos to order to inspire relatives, medical staff, and volunteers to understand people with dementia. This project has been granted financial support from the Ministry of Social Affairs in Denmark. She has conducted the first Danish investigation on the effect of sufficient daylight on the human spirit, activity level, and biological rhythm. E-mail: **villahvide@mail.dk**

**Maj Lyck Nielsen,** Occupational Therapist, specialized in psychiatry, Holistic Therapy assistant at the Quality of Life Research Center in Copenhagen, Denmark. Student at the Nordic School of Holistic Medicine. E-mail: mln@livskvalitet.org

**Joav Merrick, MD, MMedSci, DMSc,** is professor of child health and human development affiliated with the Center for Multidisciplinary Research in Aging, Zusman Child Development Center, Division of Pediatrics and Community Health at the Ben Gurion University, Beer-Sheva, Israel; the medical director of the Division for Mental Retardation, Ministry of Social Affairs, Jerusalem; and the founder and director of the National Institute of Child Health and Human Development. Dr. Merrick has numerous publications in the field of child health and human development, rehabilitation, intellectual disability, disability, health, welfare, abuse, advocacy, quality of life, and prevention and received the Peter Sabroe Child Award for outstanding work on behalf of Danish Children in 1985 and the International LEGO-Prize ("The Children's Nobel Prize") for an extraordinary contribution towards improvement in child welfare and well being in 1987. E-mail: jmerrick@internet-zahav.net. Website: www.nichd-israel.com
